# Supplementary material for: Multi‐dataset identification of innovative feature genes and molecular mechanisms in keratoconus
Source: J Cell Mol Med. 2024 Sep 19;28(18):e70079. doi: 10.1111/jcmm.70079 (PMC11412914; doi:10.1111/jcmm.70079)
Supplement: Supplementary file 2 — Material S2. [file JCMM-28-e70079-s002.zip › 7.GSEA analysis/3.DBNDD1/KEGG_CYSTEINE_AND_METHIONINE_METABOLISM.html]

Details for gene set KEGG\_CYSTEINE\_AND\_METHIONINE\_METABOLISM[GSEA]

|  || Dataset | DBNDD1.group.cls#h\_versus\_l |
| Phenotype | group.cls#h\_versus\_l |
| Upregulated in class | h |
| GeneSet | KEGG\_CYSTEINE\_AND\_METHIONINE\_METABOLISM |
| Enrichment Score (ES) | 0.2021533 |
| Normalized Enrichment Score (NES) | 0.8911126 |
| Nominal p-value | 0.6363636 |
| FDR q-value | 1.0 |
| FWER p-Value | 1.0 |
Table: GSEA Results Summary

  

Fig 1: Enrichment plot: KEGG\_CYSTEINE\_AND\_METHIONINE\_METABOLISM      
 Profile of the Running ES Score & Positions of GeneSet Members on the Rank Ordered List

  

| SYMBOL | TITLE | RANK IN GENE LIST | RANK METRIC SCORE | RUNNING ES | CORE ENRICHMENT || 1 | TAT | na | 2033 | 0.298 | 0.0561 | Yes |
| 2 | IL4I1 | na | 3376 | 0.237 | 0.1085 | Yes |
| 3 | BHMT | na | 5048 | 0.179 | 0.1293 | Yes |
| 4 | LDHAL6A | na | 6564 | 0.135 | 0.1378 | Yes |
| 5 | CDO1 | na | 6765 | 0.130 | 0.1821 | Yes |
| 6 | MTAP | na | 9469 | 0.079 | 0.1345 | Yes |
| 7 | MAT1A | na | 10221 | 0.068 | 0.1392 | Yes |
| 8 | TRDMT1 | na | 10277 | 0.067 | 0.1636 | Yes |
| 9 | MTR | na | 10492 | 0.065 | 0.1823 | Yes |
| 10 | MAT2A | na | 10641 | 0.063 | 0.2022 | Yes |
| 11 | AHCY | na | 11881 | 0.048 | 0.1850 | No |
| 12 | LDHC | na | 12007 | 0.047 | 0.1995 | No |
| 13 | GOT2 | na | 13748 | 0.032 | 0.1617 | No |
| 14 | MPST | na | 14264 | 0.028 | 0.1577 | No |
| 15 | DNMT3L | na | 14297 | 0.028 | 0.1675 | No |
| 16 | CBS | na | 16188 | 0.015 | 0.1186 | No |
| 17 | DNMT3A | na | 16223 | 0.014 | 0.1232 | No |
| 18 | SMS | na | 18350 | 0.000 | 0.0620 | No |
| 19 | ENOPH1 | na | 18569 | -0.001 | 0.0562 | No |
| 20 | AMD1 | na | 19469 | -0.008 | 0.0335 | No |
| 21 | ADI1 | na | 19848 | -0.012 | 0.0271 | No |
| 22 | GOT1 | na | 19996 | -0.013 | 0.0279 | No |
| 23 | AHCYL1 | na | 20689 | -0.019 | 0.0153 | No |
| 24 | SRM | na | 20771 | -0.020 | 0.0206 | No |
| 25 | SDS | na | 20980 | -0.022 | 0.0231 | No |
| 26 | LDHB | na | 22462 | -0.037 | -0.0054 | No |
| 27 | MAT2B | na | 22727 | -0.040 | 0.0024 | No |
| 28 | LDHA | na | 24317 | -0.061 | -0.0201 | No |
| 29 | AHCYL2 | na | 25519 | -0.079 | -0.0243 | No |
| 30 | APIP | na | 26867 | -0.105 | -0.0228 | No |
| 31 | DNMT3B | na | 27712 | -0.124 | 0.0007 | No |
| 32 | LDHAL6B | na | 28137 | -0.134 | 0.0402 | No |
| 33 | DNMT1 | na | 28434 | -0.142 | 0.0862 | No |
| 34 | CTH | na | 31662 | -0.247 | 0.0884 | No |
Table: GSEA details [plain text format]

  

Fig 2: KEGG\_CYSTEINE\_AND\_METHIONINE\_METABOLISM      
 Blue-Pink O' Gram in the Space of the Analyzed GeneSet

  

Fig 3: KEGG\_CYSTEINE\_AND\_METHIONINE\_METABOLISM: Random ES distribution      
 Gene set null distribution of ES for **KEGG\_CYSTEINE\_AND\_METHIONINE\_METABOLISM**
